# Dublin College of Surgeons—Apprenticeship

**Published:** 1831-01-01

**Authors:** 


### XLVI.

### Dublin College of Surgeons—Apprenticeship.

Our readers are aware that we have all along raised our voice against the system of apprenticeship generally. In the 24th Number of this Journal, page 446, *et seq.* we protested in strong language against a bye-law of the Irish College of Surgeons, which conferred marked *favours* on the apprentice-class of candidates for collegiate honours—

*because that class evidently put money into the purse of the College*—while the said bye-law threw various obstacles in the way of those who approached the dread tribunal, without first burning golden incense at the shrines of the MINOS, ÆACHUS, and RHADAMANTHUS of the Hibernian Tartarus. By some late proceedings in the councils of those regions, we perceive that Mr. Carmichael having made strenuous exertions to repeal the obnoxious and disgraceful by-law above alluded to, was foiled in the meritorious attempt, as might well be expected when VIRTUS was to contend with NUMMUS—and subsequently obliged, from a sense of honour and propriety, to voluntarily withdraw himself from the Court of Assistants of the College. The reader will form a tolerable estimate of the causes of this resignation from Mr. Carmichael's letter to the president, as published in a late Number of the LANCET.

"Rutland Square, Nov. 1st, 1830.

"Sir,—I deeply regret that the by-laws respecting the qualifications of candidates for letters testimonial, have been confirmed by the College at its last meeting, although, during a protracted discussion of several days, sufficient facts and reasonings were adduced to convince any unprejudiced mind of their partiality, injustice, and direct violation of the spirit of our charter. I therefore, Sir, feel myself under the necessity of resigning my seat as a member of the Court of Assistants; for, to hold office after the failure of every possible exertion to repeal those obnoxious by-laws, would be justly considered a tacit approval of their continuance, and of the councils by which the College has been for some time directed, and which, to say the least, I cannot consider but as short-sighted, imprudent, and directly opposed to the honour and dignity of the College, which every member on admission pledges himself publicly and solemnly to uphold to the best of his abilities.

Our new charter has opened two distinct roads by which a license to practise surgery in Ireland can be obtained; the one by apprenticeship and conformity to a certain system of education, which, in the words of the charter, is to be 'hereafter laid down by the College;' the other merely consists in a conformity to the system of education, but without an apprenticeship. Now the College has, in my apprehension, unfairly taken advantage of this unfortunate word '*hereafter*,' and instead of laying down one system of education for both classes of pupils, has enacted a distinct system for each, and this is done in such a manner as must convince any disinterested person who peruses the by-laws relating to this subject, that the object of the College is to discourage all pupils from entering into the profession by any other route than that of an apprenticeship. It therefore follows that the non-indented candidate will enter on the ordeal of an examination, with an impression that he is about to stake his reputation and future prospects in life before a prejudiced tribunal, against whose prepossessions he can have no other dependence save the publicity of his examination, which wise precaution, however needful, will not remove from his mind a conviction, that he goes to trial before judges impressed with a belief that they will serve themselves by his rejection.

It was for the purpose of removing altogether these objections, that I moved for a committee to reconsider the by-laws in question, for I shall venture to assert that there is not a member of the College who has more at heart its true interests than myself, and it is with regret that I feel myself compelled to resign all connexion by office with a body to which I must naturally be attached, were it only by habit, being a licentiate or member for upwards of thirty years, during twenty-six of which I successively held office either as a member of the Court of Examiners, or Court of Appeal, or as vice-president, or president of the College.

I have the honour to be, Sir,
Your most obedient,
and very humble servant,
RICHARD CARMICHAEL."

It is needless to make any comment on the subject. The Irish medical aristocracy is running the same race as the aristocracy in general on both sides of the Channel! The fruits will be seen in good time.

